# Supplementary material for: Actuating compact wearable augmented reality devices by multifunctional artificial muscle
Source: Nat Commun. 2022 Jul 18;13:4155. doi: 10.1038/s41467-022-31893-1 (PMC9293895; doi:10.1038/s41467-022-31893-1)
Supplement: Supplementary file 1 — Supplementary Information [file 41467_2022_31893_MOESM1_ESM.pdf]

Supplementary Information

Actuating Compact Wearable Augmented Reality Devices by Multifunctional Artificial Muscle

Dongjin Kim[1†], Baekgyeom Kim[1†], Bongsu Shin[2,3†], Dongwook Shin[1], Chang-Kun Lee[2,3], Jae-Seung Jung[2,3], Juwon Seo[2,3], Yun-Tae Kim[2,3], Geeyoung Sung[2,3], Wontaek Seo[2], Sunil Kim[2], Sunghoon Hong[2], Sungwoo Hwang[2,4], Seungyong Han[1*], Daeshik Kang[1*], Hong-Seok Lee[2,5*] and Je-Sung Koh[1*]

[1]Department of Mechanical Engineering, Ajou University, 206 Worldcup-ro, Yeongtong-gu, Suwon-si, Gyeonggi-do, 16499, Republic of Korea

[2]Samsung Advanced Institute of Technology, Samsung Electronics, 130 Samsung-ro, Yeongtong-gu, Suwon-si, Gyeonggi-do, 16678, Republic of Korea

[3]Samsung Electronics, 34, Seongchon-gil, Seocho-gu, Seoul, 06765, Republic of Korea

[4]Samsung SDS, 125, Olympic-ro, 35-gil, Songpa-gu, Seoul, 05510, Republic of Korea

[5]Seoul National University, 1, Gwanak-ro, Gwanak-gu, Seoul, 08826, Republic of Korea

*Address correspondence to: sy84han@ajou.ac.kr, dskang@ajou.ac.kr, lhs1210@samsung.com, jskoh@ajou.ac.kr

† Equal contribution

This PDF file includes:

- Supplementary Note 1 to 2
- Supplementary Figure 1 to 21
- Supplementary Table 1 to 6

Other Supplementary Videos for this manuscript includes the following:

- Supplementary Movie 1 to 10

Supplementary Note 1. Scaling up the actuation strain and force

Here, we describe a method to scale up the actuation stroke and force of the CASA-based actuator, which can be easily designed owing to the simple structure and easy fabrication of the CASA.

To extend the actuation stroke, CASAs are connected serially using adhesive in the actuation direction, as shown in Supplementary Fig. 10a. As shown in Supplementary Fig. 10b, serially connected CASAs are embedded in the origami linear stage using Sarrus linkage with a 60 g load. This origami linear stage is used for the linear motion of the actuator. When voltage is applied to the serially connected CASAs, the actuator is deformed, as shown in Supplementary Fig. 10c and Movie 5. By serially connecting CASAs, seven times larger actuation stroke was achieved than that of a single CASA.

To amplify the actuation force, the SMA wire is embedded along the dashed line, as shown in Supplementary Fig. 10d. The actuation strain of this actuator is similar to that of the CASA with two lines of embedded SMA wire. However, owing to the additional lines of the SMA wire, this actuator can generate force several times more than the CASA with one or two rows of embedded SMA wire. This force-amplified CASA can lift approximately 800 times heavier weight than its own, as shown in Supplementary Fig. 10e and 10f and Movie 6.

Supplementary Note 2. Impulsive actuation and consumed energy of BPS

The actuation time of CASA with BPS is reduced by applying an impulsive electric current. This impulsive actuation enables a fast state transition between stable states. To verify the possibility of impulsive actuation, we performed a parametric study on input electric current and actuating time as shown in Supplementary Fig. 13. The electric power applied to the CASA and counter SMA wire can be calculated from average voltage and current as shown in Supplementary Fig. 14a. Although the power increases upon increasing the electric current, the total energy input for actuation gradually decreases upon applying impulsive electric current in the CASA and counter SMA wire as shown in Supplementary Fig. 14b and c.

The state transition time of BPS is proportional to the applied voltage time. The impulsive actuation has the advantages of rapid transition of state and minimum electric current. Supplementary Figure 14b and c show the input energy calculated from the applied voltage and current. As the high current is applied for a short time, the input energy is close to the theoretical heat energy. Here, the amount of theoretical heat required to raise the temperature is calculated follows:

$$Q = mc_p\Delta T$$

where Q is the amount of energy, m is the mass of the SMA wire, c_p is the specific heat capacity of the SMA wire, and ΔT is the temperature rise of the SMA wire. The dimension and thermal properties of the SMA wire are described in Supplementary Table 5. Comparing the calculated input energy and theoretical heat energy, we confirm that adjusting the actuation time can reduce energy wastage due to heat convection. Moreover, the CASA with BPS maintain its position without electric energy. This means that no energy is consumed to maintain near or far depth in AR applications.

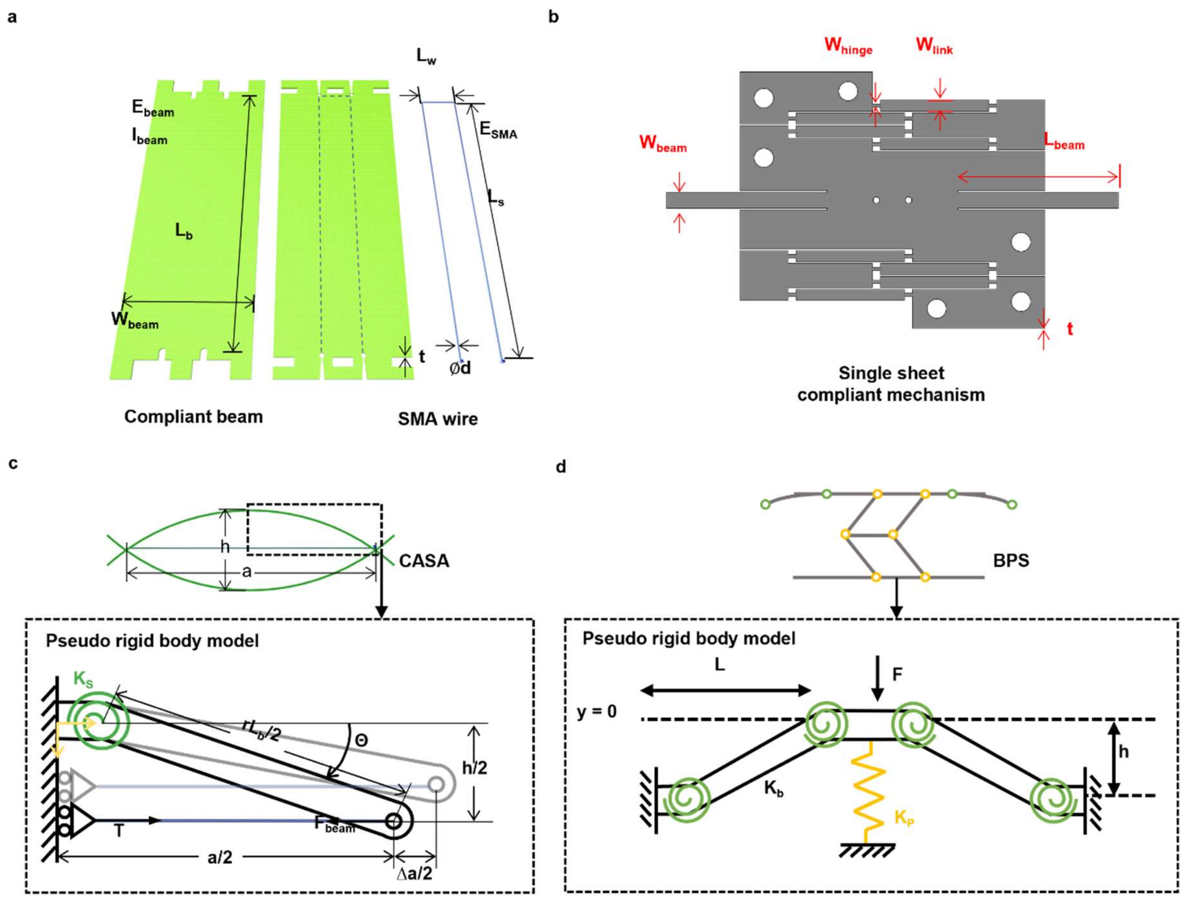

Supplementary Figure 1. Design parameters and Pseudo-rigid-body models of CASA and BPS. a, Top-view illustration and parameters of CASA. **b,** Top-view illustration and parameters of BPS. **c** and **d,** Pseudo-rigid-body model parameters of CASA (**c**) and BPS (**d**).

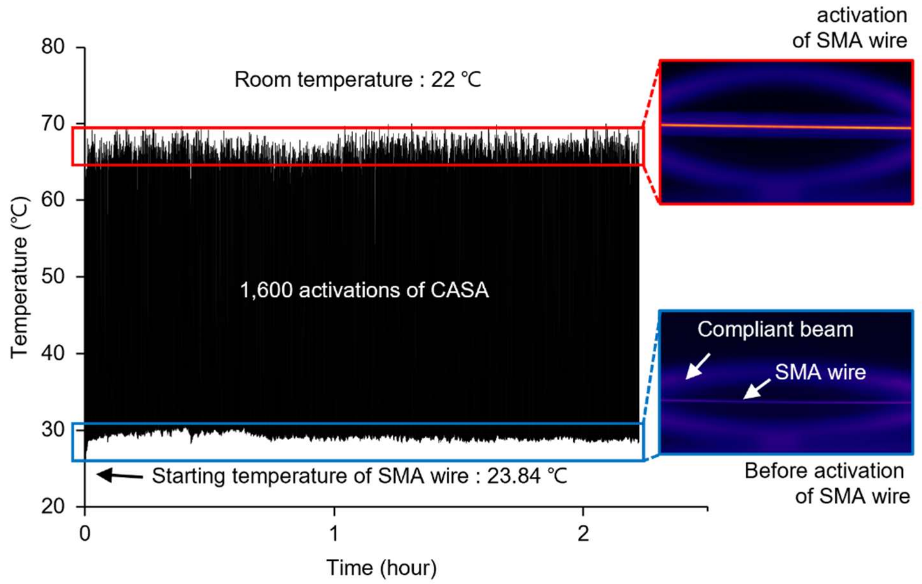

Supplementary Figure 2. **Temperature of the SMA wire during cyclic actuation of the CASA.** Temperature variation of the SMA wire measured using an infrared camera when the CASA is repeatedly actuated. The diameter of the SMA wire used in the experiment is 0.1 mm. The SMA wire is heated by Joule heating for 1 second and has a cooling time of 4 seconds in one actuation cycle.

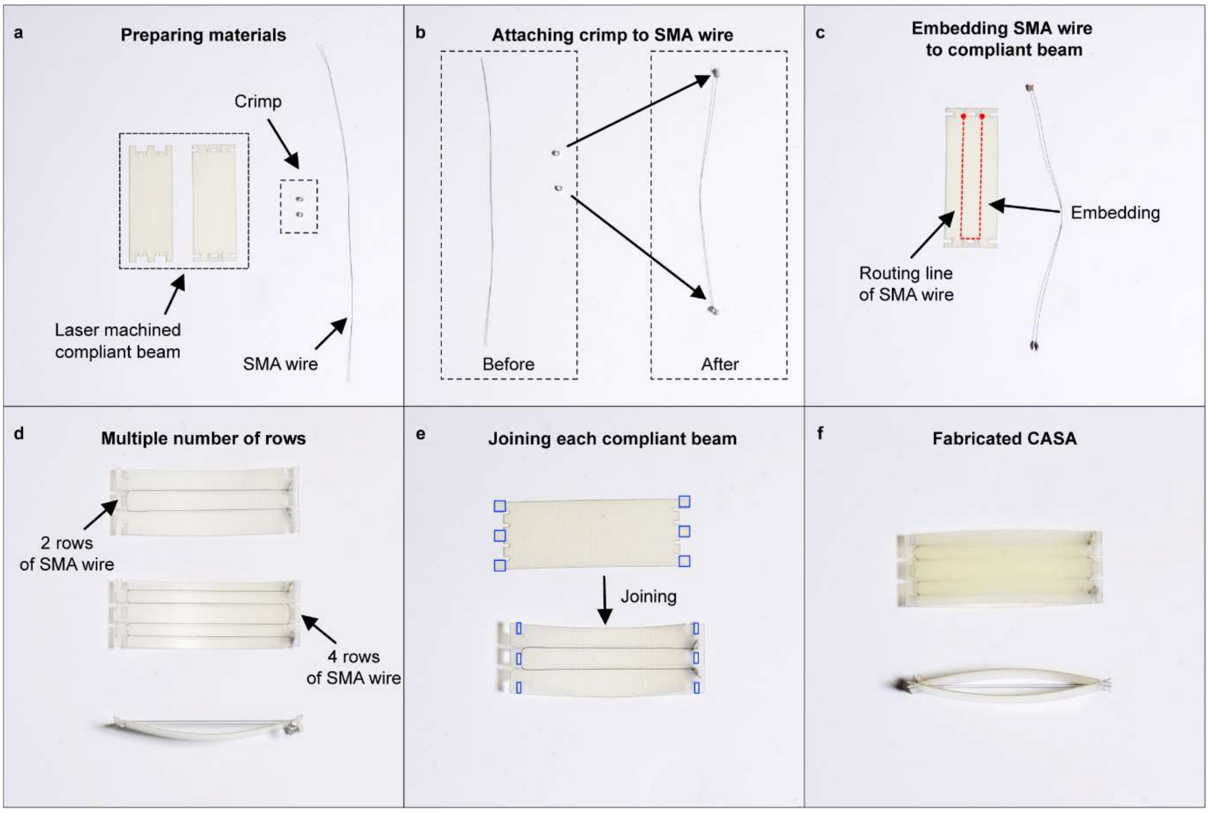

Supplementary Figure 3. Fabrication process of CASA. a, preparation of the material for fabricating CASA including laser machined compliant beam, crimps and SMA wire. **b,** Before and after attaching crimp to SMA wire. **c,** Embedding SMA wire to compliant beam along the routing line. **d,** Multiple number of rows for increasing actuation force. **e,** Joining each compliant beam. **f,** Optical image of fabricated CASA.

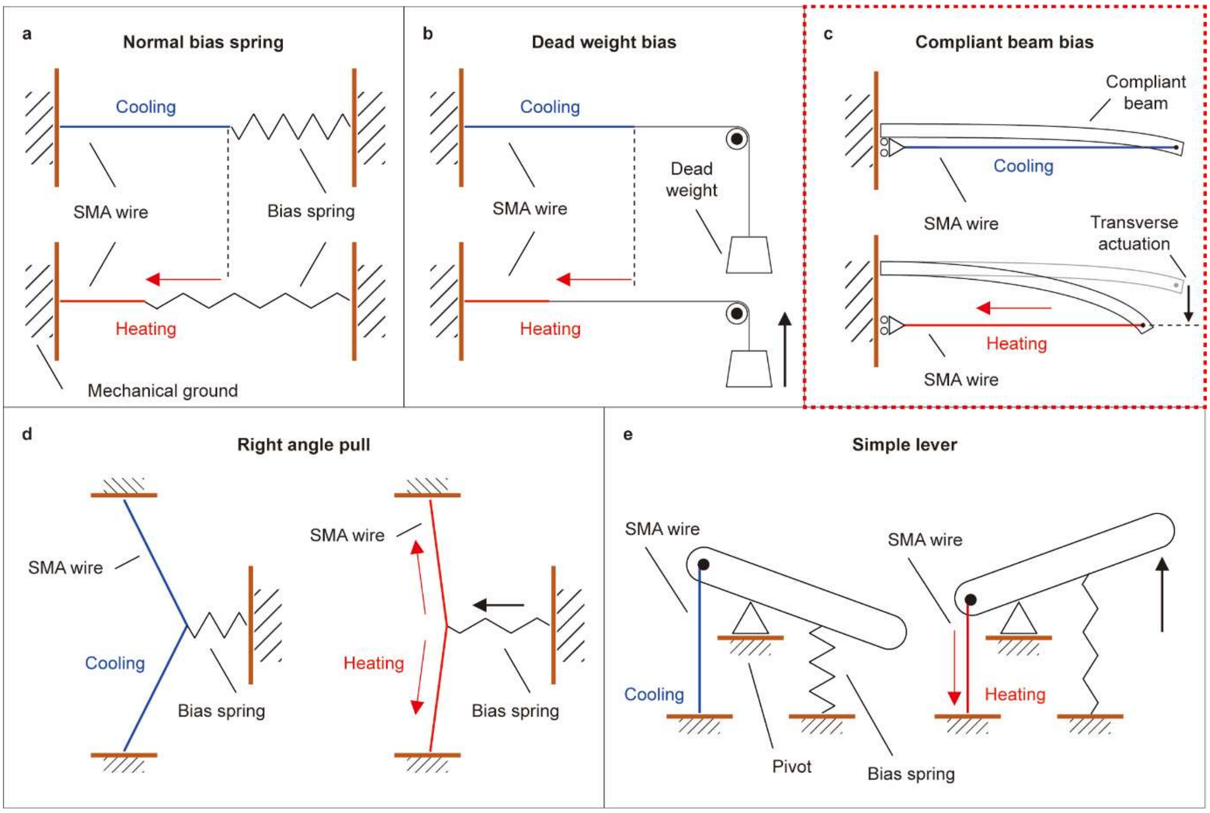

Supplementary Figure 4. **Comparison of variable amplification structure.** Type of (**a**) Normal bias spring, (**b**) Dead weight bias, (**c**) Compliant beam bias type, (**d**) Right angle pull, and (**e**) Simple lever.

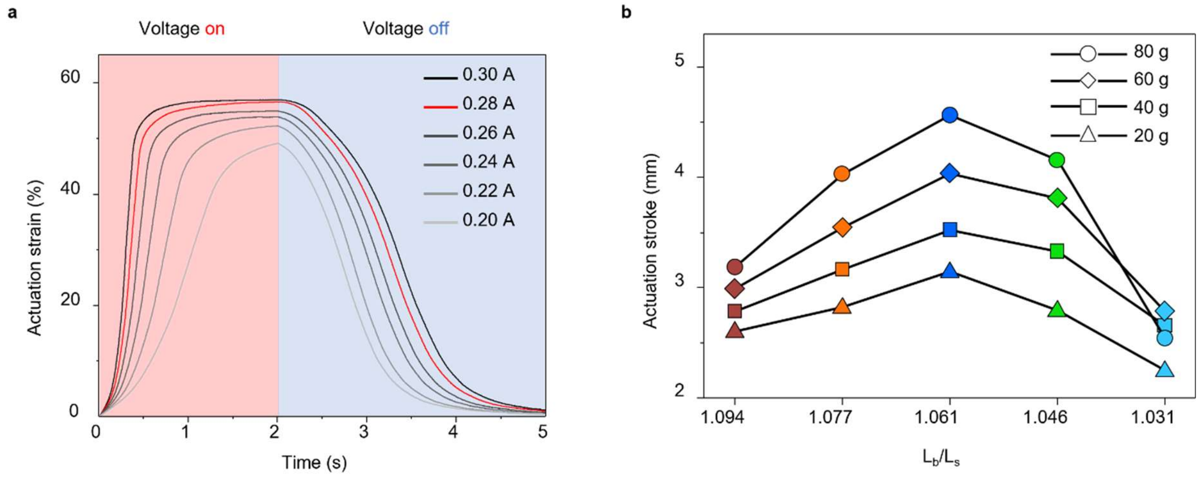

Supplementary Figure 5. Experimental results of the CASA. a, Actuation strain of CASA for different applied voltages, where current was applied for 2 s. **b,** Actuation stroke of CASA with different initial SMA wire lengths under different preload weights.

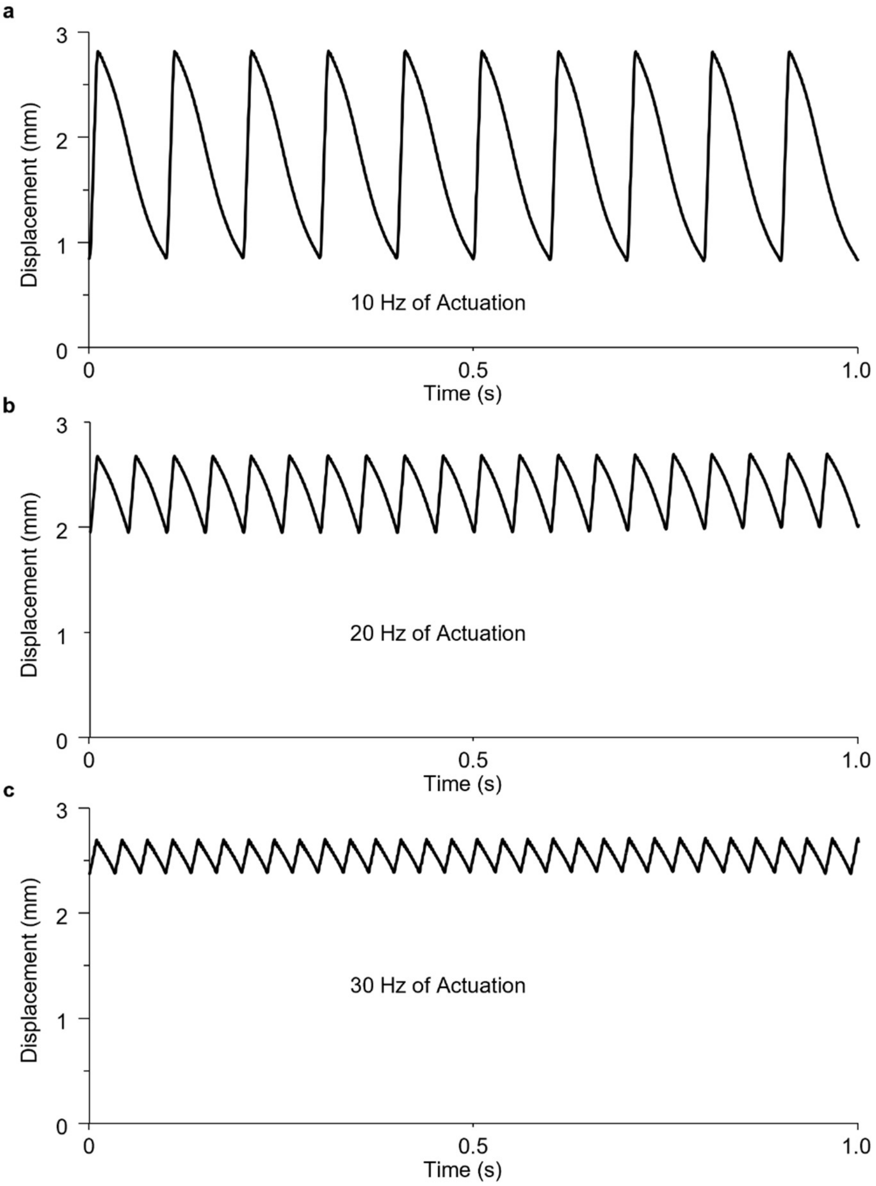

Supplementary Figure 6. **Actuation displacement as a function of frequency for SMA wire with a diameter of 0.025 mm. a,** Actuation displacement of CASA under 10 Hz **b,** 20 Hz **c,** 30 Hz.

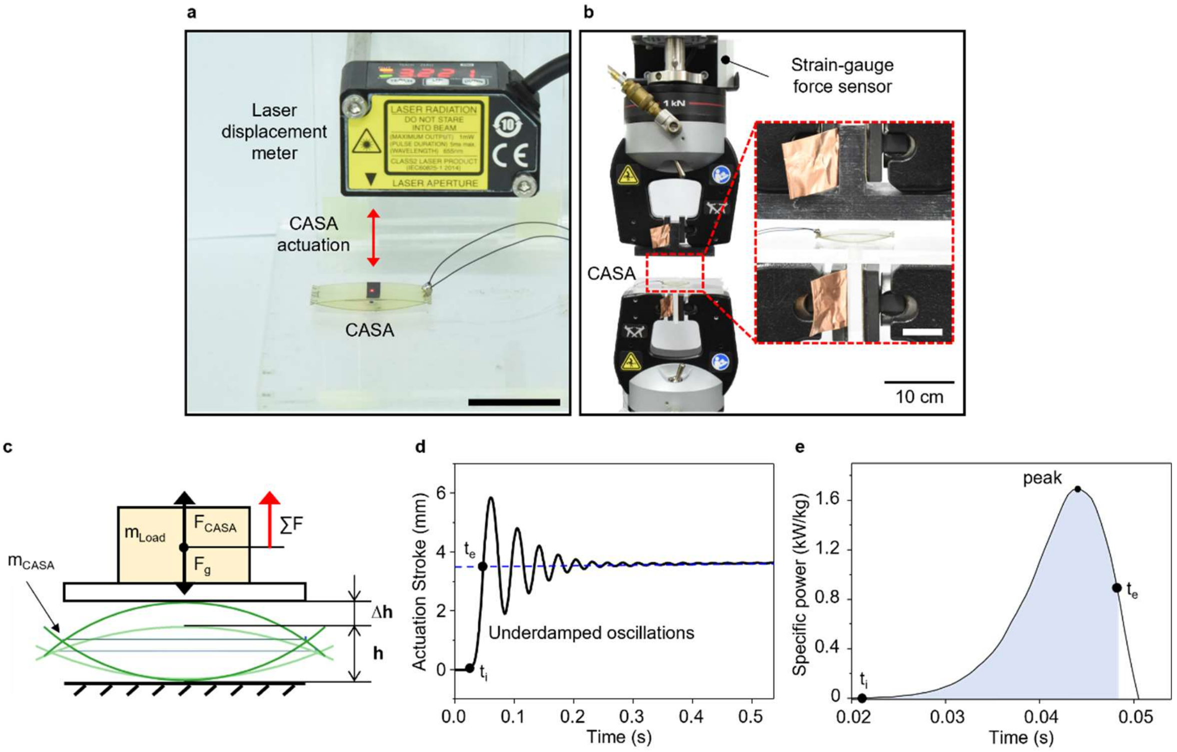

Supplementary Figure 7. Performance characterization of CASA. a, Experimental setup for measuring the actuation stroke of the CASA. **b,** Experimental setup for measuring the blocked force of CASA. **c,** Schematic of the experimental setup for measuring the actuation stroke of the CASA with additional load. **d,** Actuation stroke of pre-loaded CASA when voltage is applied. t_i and t_e correspond to the times of initial and equilibrium actuation, respectively. **e,** Specific power plot of pre-loaded CASA.

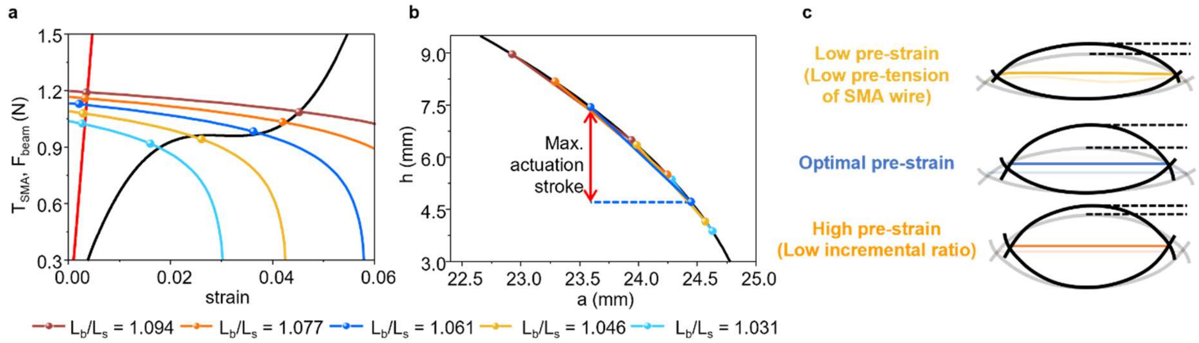

Supplementary Figure 8. Analytic modeling result of CASA. a, Force-strain curve plots of SMA and compliant beam. **b,** Vertical displacement as a function of horizontal displacement of CASA.

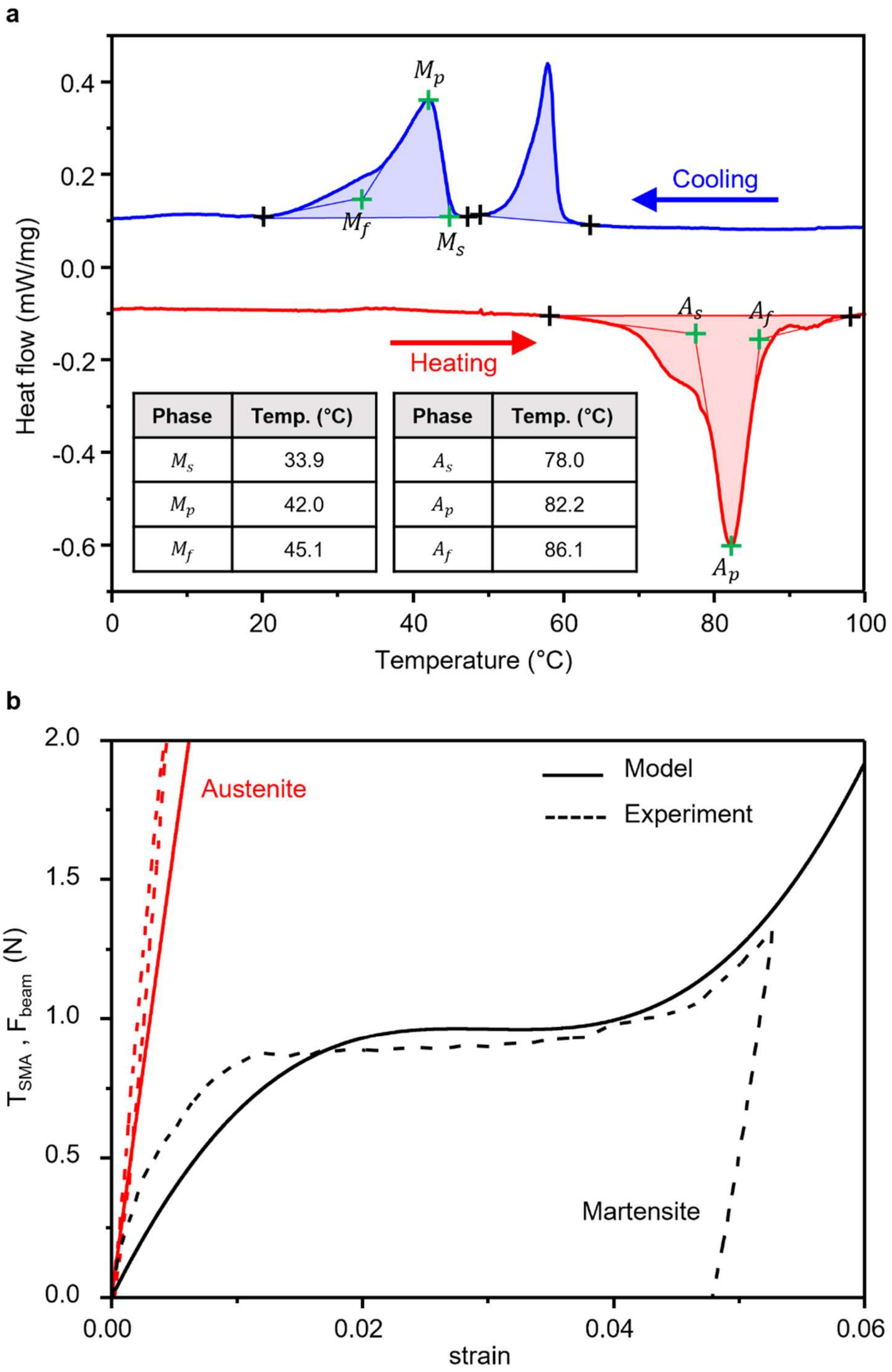

Supplementary Figure 9. **Thermomechanical properties of SMA wire**. **a,** Differential scanning calorimetry (DSC) results showing transition temperature of SMA wire (A_s, A_f, M_s and M_f). **b,** Analytical model predictions and experimental results for force vs. strain relationship of the SMA wire. Red and black colour represent austenite and martensite phase, respectively.

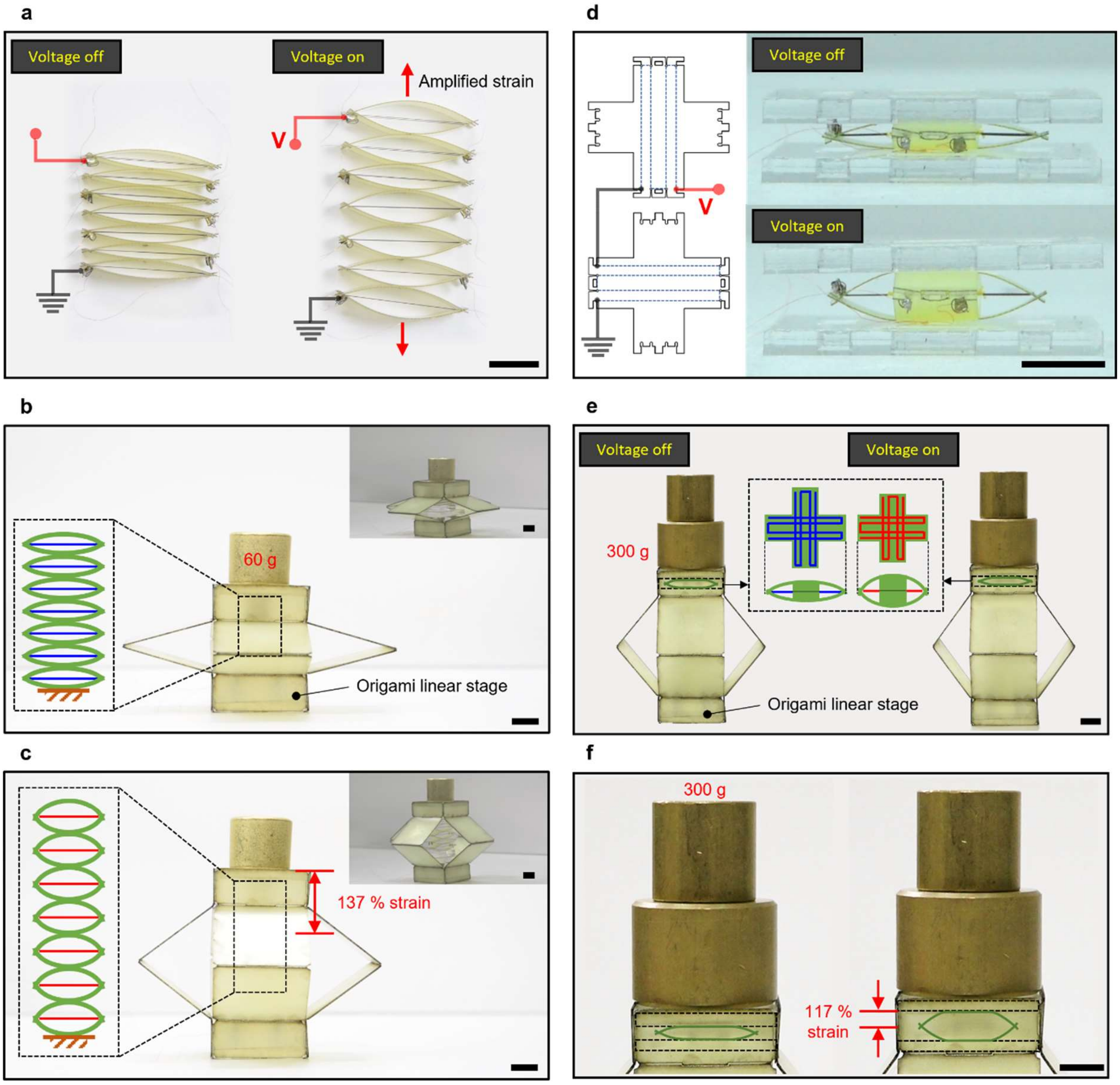

Supplementary Figure 10. Maximization of actuation stroke and force. a, Serially connected CASA for maximizing actuation stroke before and after applying voltage. **b** and **c,** Front view of nondeformed (**b**) and deformed (**c**) serially connected CASA and origami linear stage using Sarrus linkage. (left) Schematic of the serially connected CASA embedded in the linear stage. (inset) Isometric-view optical image for exhibiting the serially connected CASA in the stage. **d,** (left) Illustrations of the CASA with additional lines of the SMA wire for amplifying the actuation force. (right) Front view of the operational image of the CASA-amplifying force. **e** and **f,** Actuation image of the CASA-amplifying force with a 300 g load.

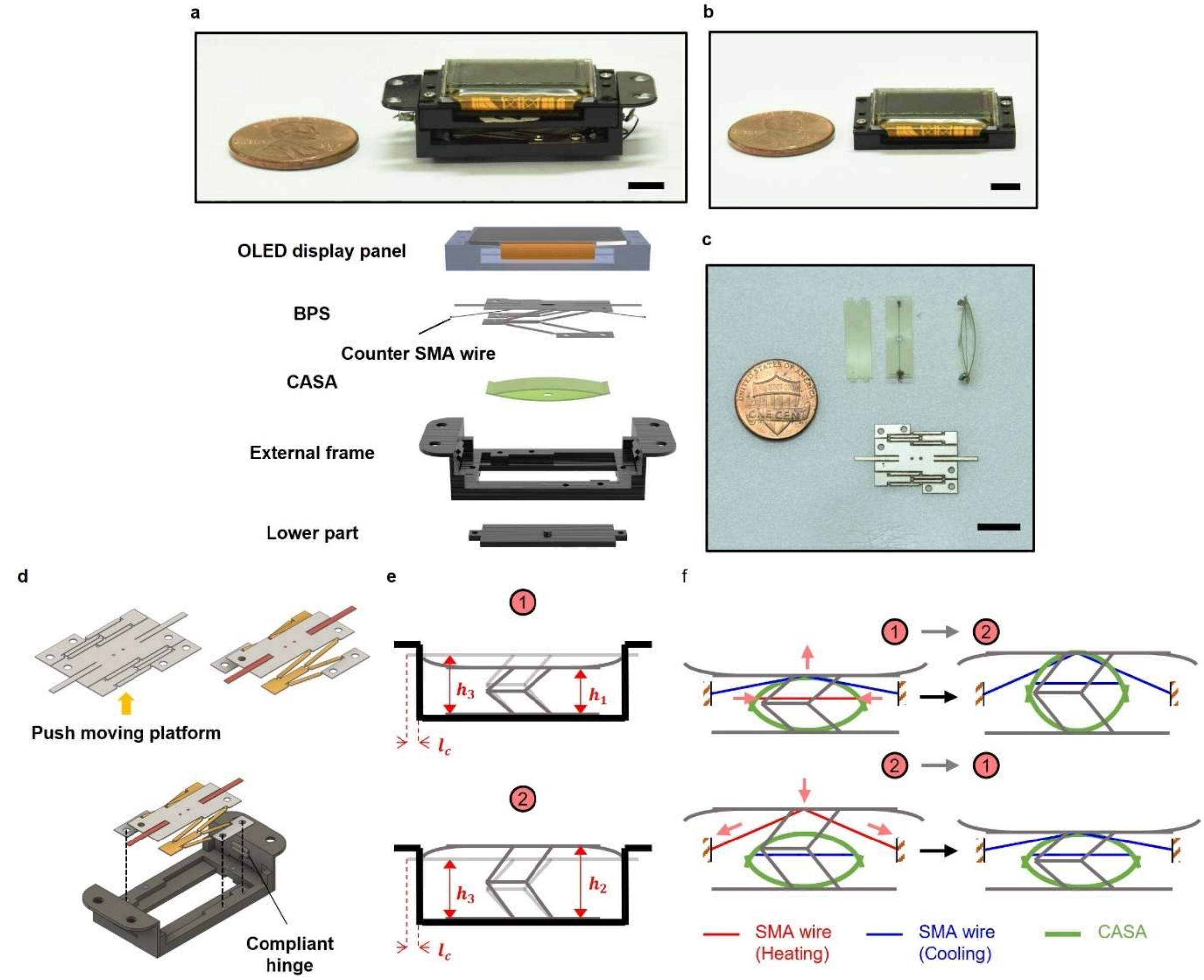

Supplementary Figure 11. Fabrication of CASA and BPS in actuator module for AR glass devices. a, Optical image and exploded-view schematic illustration of a linear actuator module for optical actuation component. **b,** Optical image of the OLED display. **c,** Top-view optical image of BPS and CASA before assembly. **d,** Fabrication procedure for inducing bi-stability of the BPS. **e,** Two stable states of the BPS with an external frame. **f,** Actuation with state transition from 1 to 2 by embedded CASA (green line) and 2 to 1 by the counter SMA actuator (diagonal).

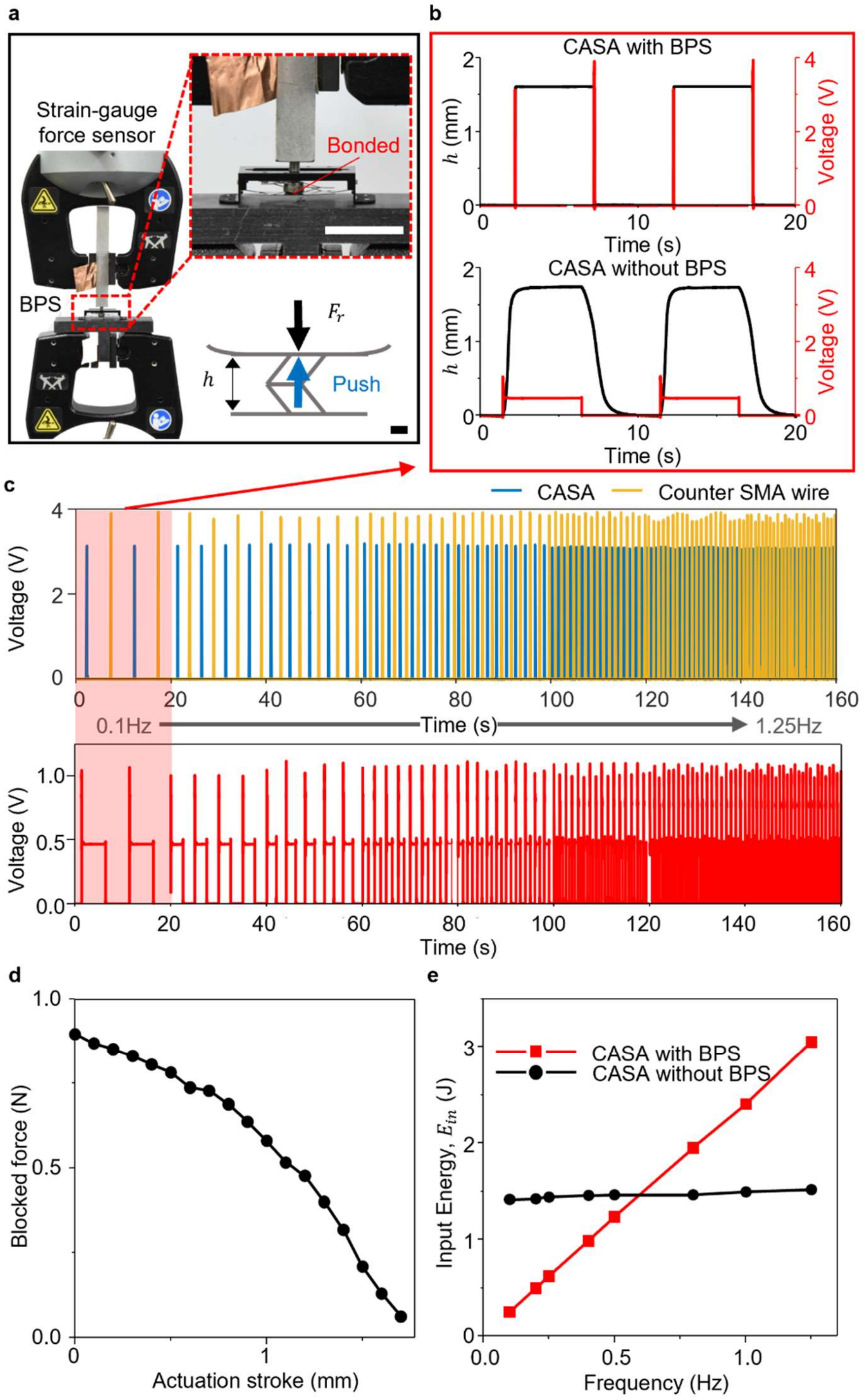

Supplementary Figure 12. Performance characterization of BPS. a, Experimental setup for measuring the reaction force of BPS. **b,** Sample experimental measurement (0.1 Hz) in Fig. 3F of actuation stroke and input voltage of the CASA with and without BPS. **c,** Actuation voltage of the CASA with BPS and Counter SMA wire (upper plot) and CASA without BPS (lower plot) with increasing the actuation frequency corresponding to Fig. 3f. **d,**

Blocked force plot of CASA with one line of embedded SMA wire. **e,** Input energy (E_{in}) plots of CASA with (red) and without (black) BPS with actuation frequency varying from 0.1 to 1.25 Hz for 20 s.

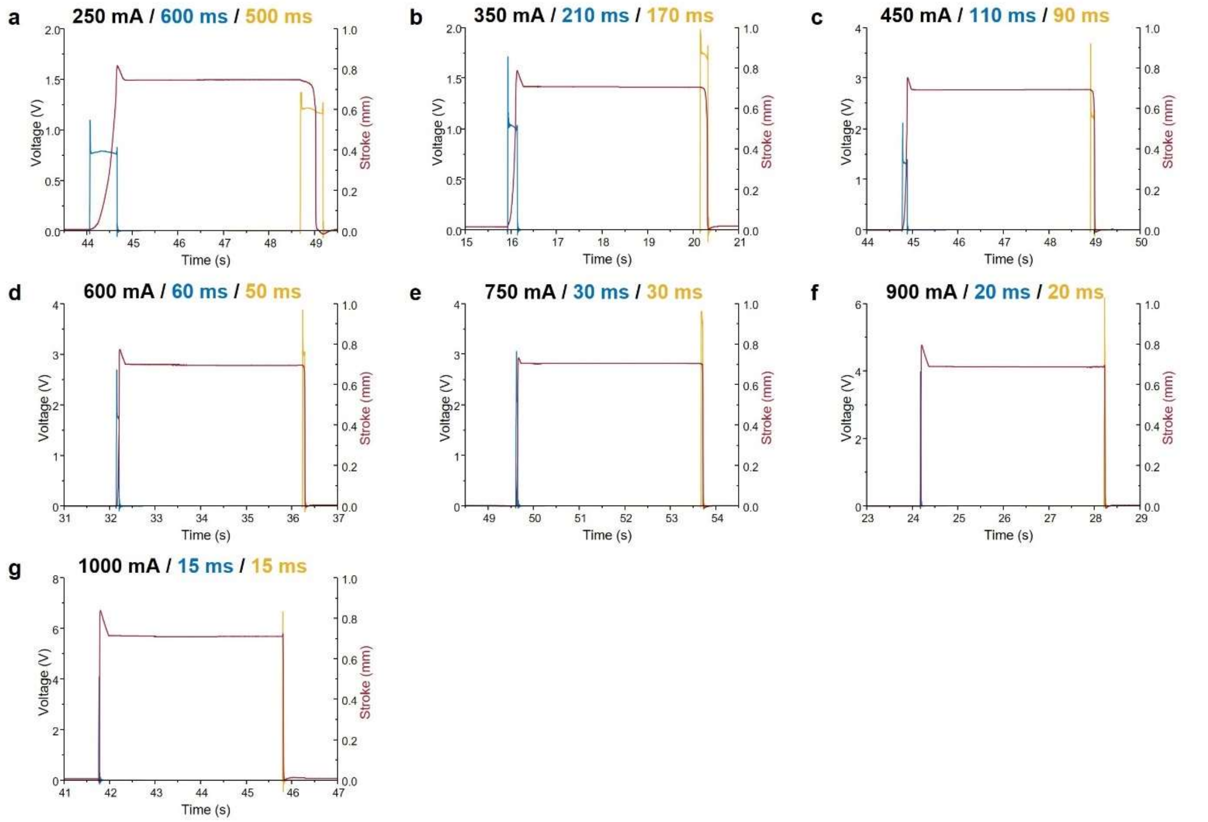

Supplementary Figure 13. Parametric study on current and actuating time. a-g, Applied voltage and actuation stroke with changing electric current and actuating time (actuating time of CASA and the counter SMA wire are denoted in blue and yellow, respectively.).

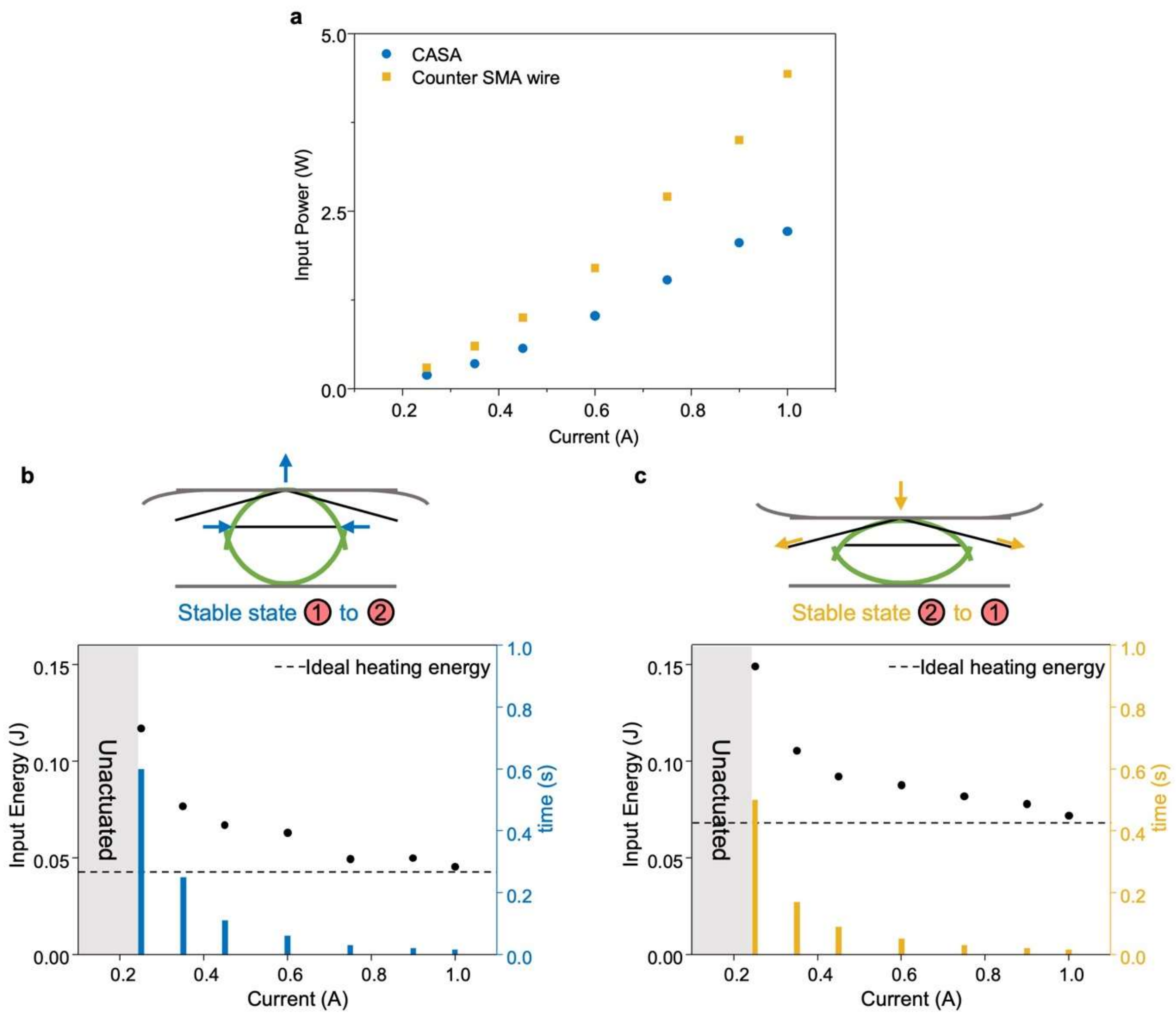

Supplementary Figure 14. **Input power and energy with varying electric current and actuation time. a,** CASA input power with changing current and actuating time. **b,** CASA input energy with changing current and actuating time. **c,** Counter SMA wire input energy with changing current and actuating time.

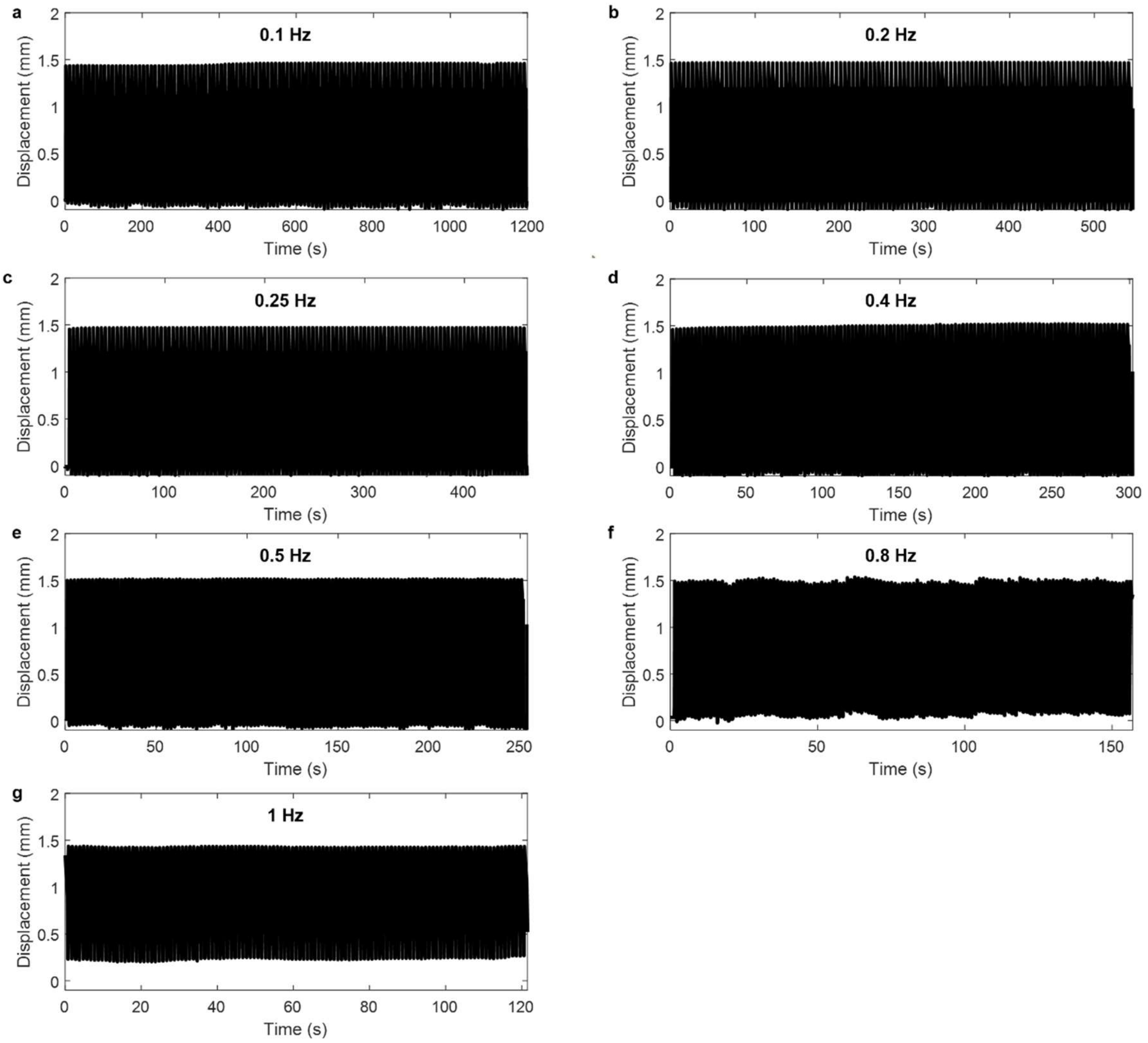

Supplementary Figure 15. **Displacement vs. time for different actuation frequencies.**

Cyclic actuation of BPS with CASA and counter SMA wire by varying frequency, **a,** 0.1 Hz, **b,** 0.2 Hz, **c,** 0.25 Hz, **d,** 0.4 Hz, **e,** 0.5 Hz, **f,** 0.8 Hz, and **g,** 1 Hz.

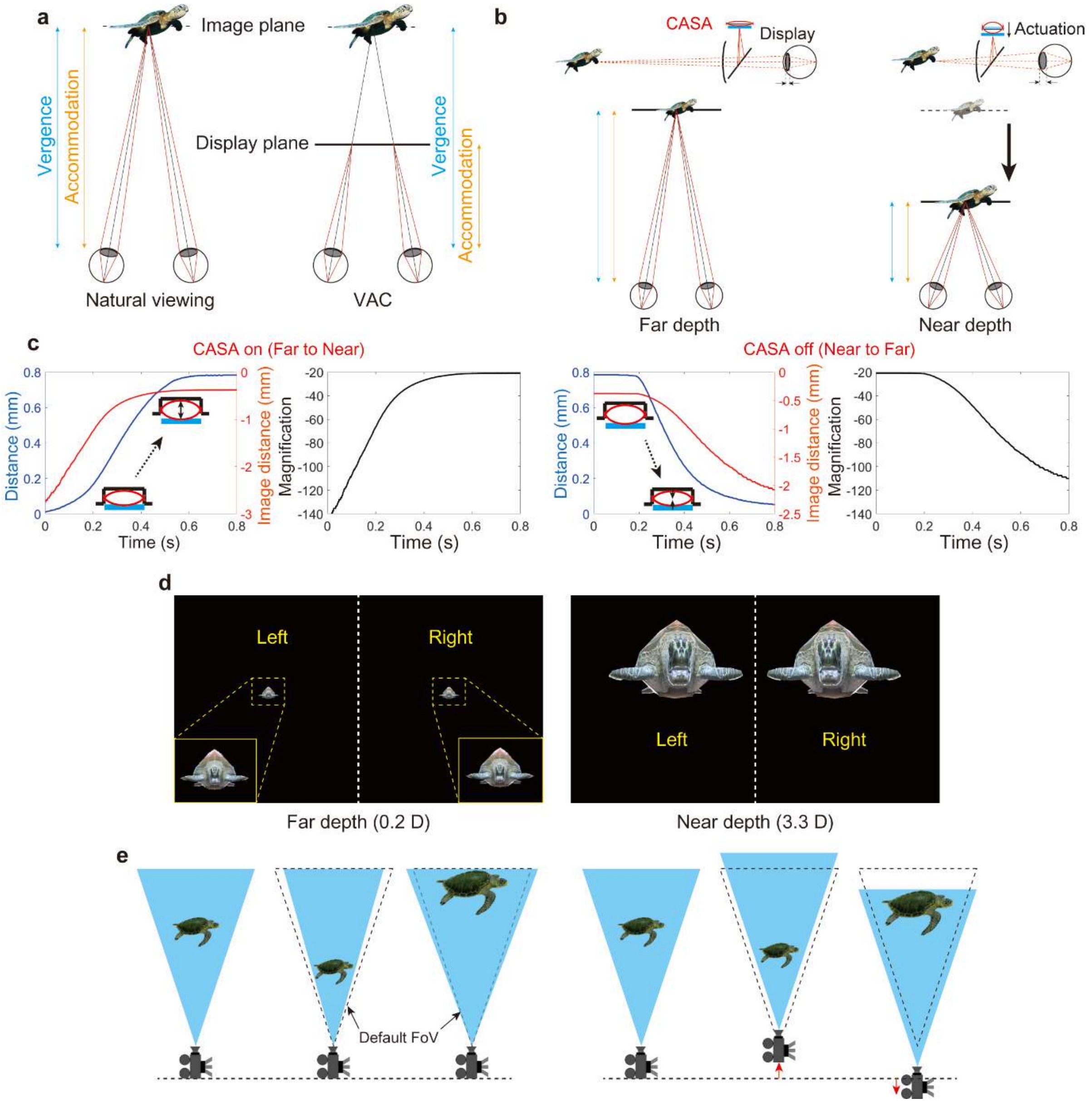

Supplementary Figure 16. Direct display switching due to CASA and 3D image rendering. a, VAC in a conventional 3D stereoscopic display. **b,** VAC reduction performed by direct display switching using CASA. **c,** Experimental data of image depth switching according to CASA operation. Negative sign of image distance means that the image is virtual because the display is located within the focal length of the curved mirror. **d,** Left and right images for binocular parallax at near/far depth. **e,** Magnification correction for 3D image-rendering method corresponding to change of depth. The default field of view of the AR prototype is determined by the optical power. The nonlinear change of the image

magnification was measured through the experiments, and the distortion of the image was corrected by reflecting it in 3D rendering. 3D rendering is performed by determining the field of view of the camera or the corrected camera depth by adding a magnification correction value to the camera position value according to the floating depth of the image.

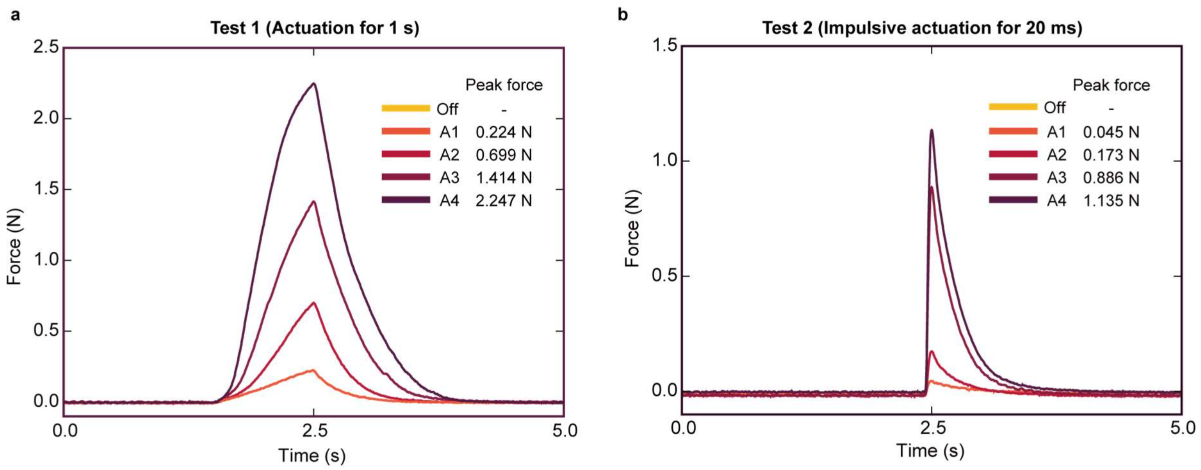

Supplementary Figure 17. **Applied force profile of the haptic test. a,** Force profiles (Off, A1, A2, A3, and A4) applying to participants in Test 1 (Actuation for 1 sec). **b,** Force profiles (Off, A1, A2, A3, and A4) applying to participants in Test 2 (Actuation for 20 ms).

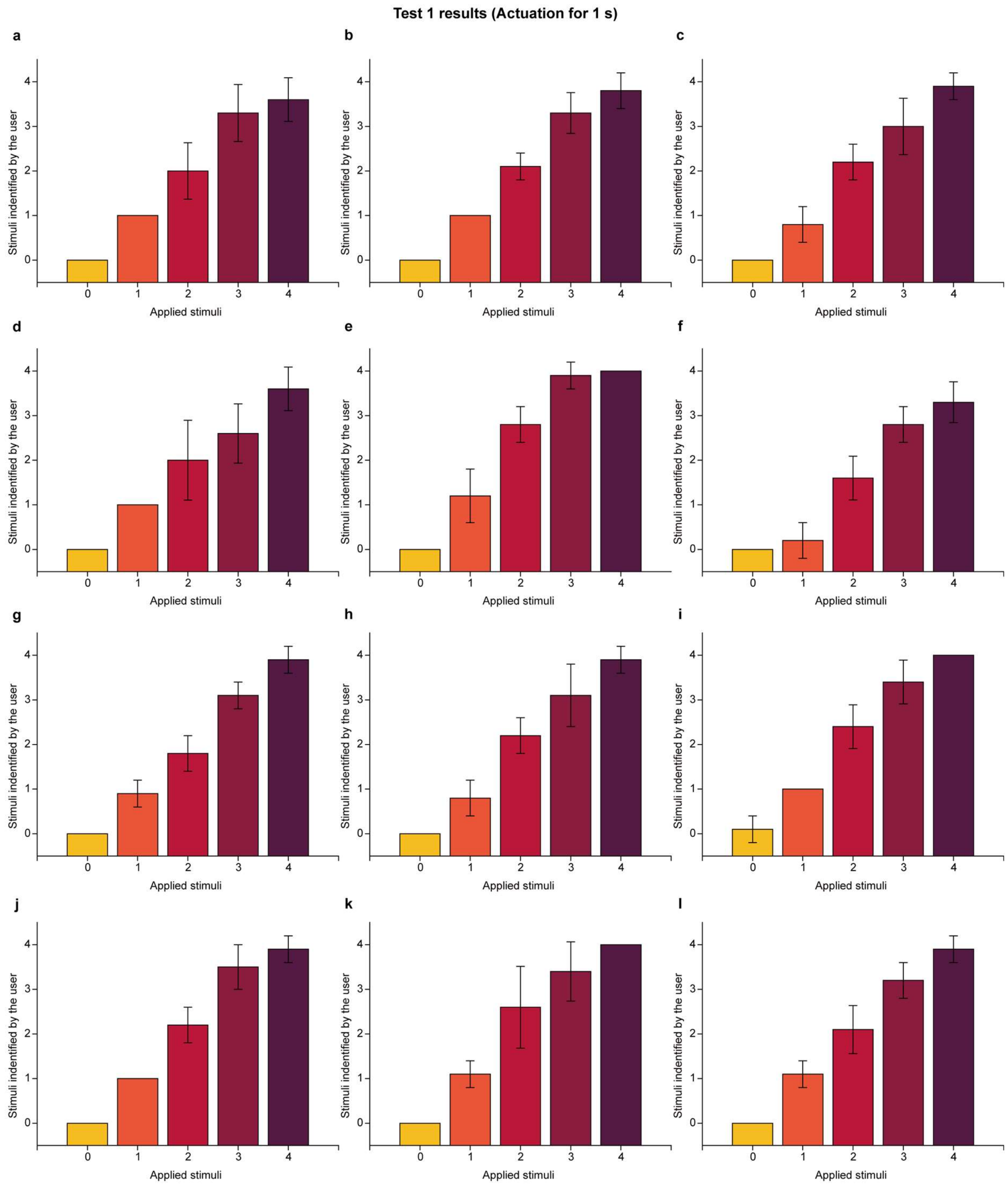

Supplementary Figure 18. **Test 1 results.** Average reported felling of the user versus applied stimuli (i.e., Test 1) for all 12 users. Error bars show mean ± standard deviation.

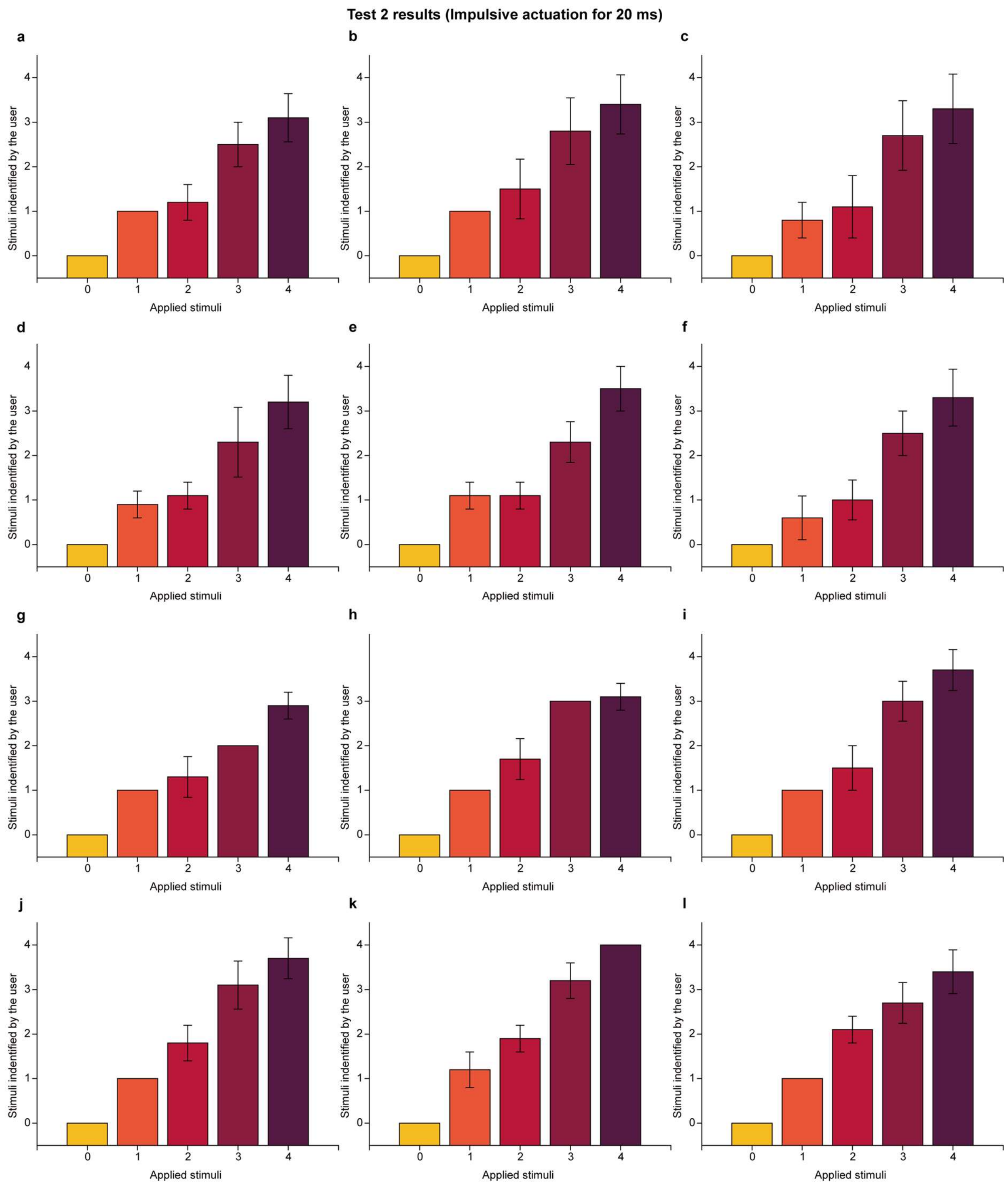

Supplementary Figure 19. **Test 2 results.** Average reported felling of the user versus applied stimuli (i.e., Test 2) for all 12 users. Error bars show mean ± standard deviation.

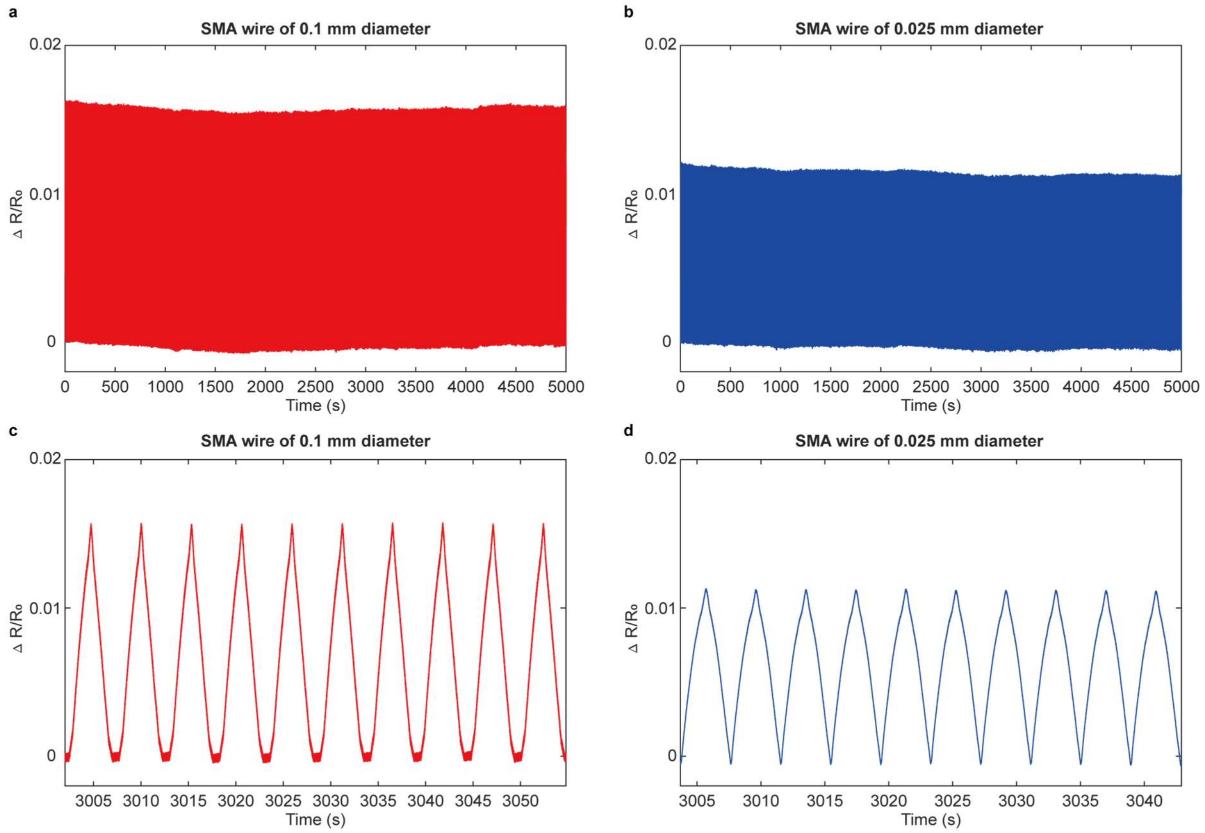

Supplementary Figure 20. **Cyclic test results of loading–unloading loop for 5,000 s. a,** 0.1 mm diameter of the SMA wire. **b,** 0.025 mm diameter of the SMA wire. **c,** Cyclic test results of 0.1 mm diameter of the SMA wire for 10 cycles **d,** Cyclic test results of 0.025 mm diameter of the SMA wire for 10 cycles. Cyclic test speed on (**a**) and (**b**) are 50 and 40 mm/min, respectively.

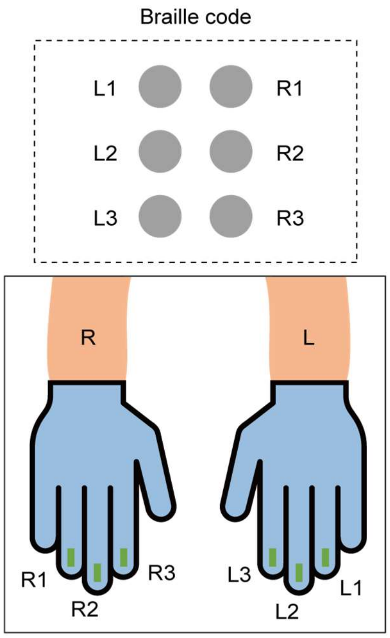

Supplementary Figure 21. CASAs embedded in the haptic glove for actuation and sensing capability. CASAs located in L1, L2, L3, R1, R2, and R3 are corresponded to each Braille code.

Supplementary Table 1. Parameters for the design of CASA.

Parameter	Symbol	Value
Mass of CASA	m_{CASA}	0.22 g
Length of the beam	L_b	12.5 mm
Characteristic radius factor	r	0.8156
Length of a row of SMA wire	L_S	22.85 mm, 23.2 mm, 23.55 mm, 23.9 mm, 24.25 mm
stiffness coefficient of the beam	K_θ	2.566
Flexural modulus of the beam	E	9.5 GPa
moment of inertia of the beam	I	0.0089 mm^4
cross-sectional area of the SMA wire	A	0.008107 mm^2
Austenite young's Modulus	E_{aus}	40 GPa
Martensite young's Modulus	E_{mar}	11.5 GPa
Residual strain	ε_L	0.0355
Critical strain	ε^{cr}	0.055

Supplementary Table 2. Comparison of variable amplification structure.

	Strain	Force
Normal bias spring	3 %	High
Dead Weight bias	4 %	High
**CASA<br>(Compliant beam bias)**	**56 %**	**High**
Right angle pull	14 %	Low
Simple lever (6:1 ex)	40 %	Low

Supplementary Table 3. Data sheet of the SMA wires.

Diameter Size (mm)	Resistance per unit length (Ohm/m)	Heating pull force (mN)	Cooling deformation force (mN)	Approximate current for 1 second contraction (mA)	Cooling time @ 90 ℃ (seconds)
0.025 (1 mil)	1425	87.2	35.3	45	0.15
0.050 (2 mil)	500	352.8	137.2	85	0.3
0.10 (4 mil)	126	1401.4	558.6	200	0.9

Supplementary Table 4. Parameters for the design of BPS.

Parameter	Symbol	Value
Mass of BPS	m_{BPS}	0.1 g
Width of buckling beam	W_{beam}	1 mm
Length of buckling beam	L_{beam}	10 mm
Thickness of SUS 304	t	150 µm
Width of parallelogram hinge	W_{hinge}	200 µm
Length of parallelogram hinge	L_{hinge}	500 µm
Width of parallelogram hinge	W_{link}	700 µm
Contraction length of bucking beam	l_c	50 µm
Young's modulus of SUS 304	E	193 GPa
Elastic beam stiffness of BPS	k_b	2.91×10^5 N/m
Parallelogram structure stiffness	k_p	410 N/m

Supplementary Table 5. Thermal characteristics of SMA wire in the actuation module.

Parameter	Symbol	Value
Weight of SMA wire in CASA	m_{CASA}	0.7852 mg
Weight of counter SMA wire	$m_{coutner}$	1.2563 mg
Specific heat of SMA wire	c_p	0.836 J/g · ℃
Density of SMA wire	ρ_{SMA}	6.45 g/cm^3
Diameter of SMA wire	W_{hinge}	0.1 mm
Length of SMA wire in CASA	l_{CASA}	15.5 mm
Length of Counter SMA wire	$l_{countet}$	24.8 mm
Room temperature	T_{room}	25 ℃
Target temperature	T_{target}	90 ℃
Theoretical heat energy of SMA wire in CASA	Q_{CASA}	42.67 mJ
Theoretical heat energy of counter SMA wire	$Q_{counter}$	68.27 mJ

Supplementary Table 6. Sample length, actuation time, voltage and current of SMA wire.

	Sample length	Actuation time, Cooling time (Frequency)	Actuation voltage	Actuation current
Figure 2d	50 mm (Diameter of 0.1 mm)	50 ms, 9950 ms (0.1 Hz)	9.5 V	1.5 A
		50 ms, 1950 ms (0.5 Hz)	9.5 V	1.5 A
		50 ms, 950 ms (1Hz)	8 V	1.2 A
		50 ms, 617 ms (1.5 Hz)	7 V	1.1 A
		50 ms, 450 ms (2 Hz)	5.9 V	0.93 A
		50 ms, 350 ms (2.5 Hz)	5.4 V	0.85 A
		50 ms, 283 ms (3 Hz)	5 V	0.8 A
	50 mm (Diameter of 0.05 mm)	10 ms, 1990 ms (0.5 Hz)	9.5 V	0.38 A
		10 ms, 990 ms (1 Hz)	9.5 V	0.38 A
		10 ms, 490 ms (2 Hz)	8.7 V	0.352 A
		10 ms, 323 ms (3 Hz)	7.8 V	0.31 A
		10 ms, 240 ms (4 Hz)	7 V	0.28 A
		10 ms, 190 ms (5 Hz)	6.5 V	0.26 A
		10 ms, 157 ms (6 Hz)	6 V	0.24 A
		10 ms, 133 ms (7 Hz)	5.7 V	0.23 A
		10 ms, 115 ms (8 Hz)	5.4 V	0.22 A
		10 ms, 91 ms (9 Hz)	5.2 V	0.21 A
	101 mm (Diameter of 0.025 mm)	10 ms, 995 ms (1 Hz)	25 V	0.18 A
		10 ms, 195 ms (5 Hz)	21 V	0.15 A
		10 ms, 95 ms (10 Hz)	18 V	0.13 A
		10 ms, 62 ms (15 Hz)	15.5 V	0.11 A
		10 ms, 47 ms (20 Hz)	13.5 V	0.1 A
		10 ms, 38 ms (25 Hz)	12.5 V	0.09 A
		10 ms, 32 ms (30 Hz)	12.2 V	0.09 A
Figure 2e	50 mm (Diameter of 0.1 mm)	2 sec (actuation time)	1.78 V	0.28 A
	101 mm (Diameter of 0.1 mm)		3.6 V	
Figure 2f	50 mm (Diameter of 0.1 mm)	20 ms (actuation time)	13 V	2.1 A
	101 mm (Diameter of 0.1 mm)		20 V	3.15 A
Figure 2i	48 mm	2 sec (actuating time)	1.75 ~ 1.8 V	0.28 A
	49 mm			
	50 mm			
	51 mm			
	52 mm			
AR device (CASA with BPS)	18 mm (SMA in CASA)	40 ms (actuating time)	3 V	0.5 A
	27 mm (Counter SMA)	45 ms (actuating time)	4 V	0.5 A
Supplementary Figure 2	50 mm (Diameter of 0.1 mm)	1 sec (actuating time)	1.78 V	0.28 A
