# Supplementary material for: Actuating compact wearable augmented reality devices by multifunctional artificial muscle
Source: Nat Commun. 2022 Jul 18;13:4155. doi: 10.1038/s41467-022-31893-1 (PMC9293895; doi:10.1038/s41467-022-31893-1)
Supplement: Supplementary file 3 — Description of Additional Supplementary Files [file 41467_2022_31893_MOESM3_ESM.pdf]

Description of Additional Supplementary Files

Supplementary Movie 1

Adjusting depth of virtual image with depth switching module
This video shows that depth switching module in AR glasses adjusts the depth of virtual image (turtle) between far (0.2 D) and near (3.3 D) focus. Camera focus of part 1 is fixed at the far plane. When CASAs in AR glasses are undeformed, a depth of the virtual image is far. The virtual image is clear because the virtual focus and the camera focus are matched. When CASAs in AR glasses are deformed, virtual object is changed to blurred image. This blurred image is due to the difference of the camera focus and virtual image depth. The depth of the virtual image is also altered between near and far in near camera focus.

Supplementary Movie 2

High-power actuation of CASA in thin and light haptic glove
This video describes the whole characteristics of the CASA which are the light weight, thin form factor, compliant structure, amplified-strain actuation and high power density of the CASA. The hand can freely grip and move with multiple number of CASAs embedded in the thin latex glove. The CASAs are able to throw 10 g of weight high in the air.

Supplementary Movie 3

Actuation of CASA
This video shows actuation and performance of CASAs. It describes actuation of optimized design of CASA and its components which are compliant beams, SMA wire, and crimps. CASA of 0.22 g can lift up preload of 80 g and show high speed actuation performance. CASA can be also designed at small scale.

Supplementary Movie 4

High-speed actuation of CASA
This video shows high-speed actuation of CASAs. It shows that CASA can be designed thinner than the smartphone and actuate at high power to throw the table tennis ball and pendulum. Under high preload we measured the maximum power density of the CASA.

Supplementary Movie 5

Scaling up actuation stroke of CASA
This video shows serial connected CASA and its scaling up actuation stroke. Part 2 shows actuating origami linear stage (Sarrus linkage) by using serial connected CASA without load. Part 3 shows actuating origami linear stage by this actuator under 60 g load.

Supplementary Movie 6

Scaling up actuation force of CASA
This video shows compact arrays of SMA wire in CASA to scale up actuation force. As shown in part 1, actuation stroke of this actuator is similar to CASA with two lines of SMA wire. However, this force-amplified CASA can lift approximately 800 times heavier weight (300 g) than its own weight as shown in part 2.

Supplementary Movie 7

Actuation of CASA with BPS
This video describes transition mechanism of BPS between the two stable state and shows actuation mechanism of CASA with BPS at 0.5 Hz and 1 Hz. Actuated CASA pushes centerline of moving platform of BPS. When the energy induced by CASA exceed threshold energy (ΔE) of bistable structure, the state of BPS is switched from state 1 to 2. To shift the state from 2 to 1, a counter SMA wire is employed. This SMA wire pulls moving platform. Similarly, the state of BPS is switched from 2 to 1 by induced energy of counter SMA wire.

Supplementary Movie 8

Actuation of depth switching module in AR glasses
This video shows utilization of CASA and CASA with BPS as depth switching module in AR glasses prototype. The two-depth switching module pushes the OLED display panel toward curved mirror. When the OLED display panel is close to the curved mirror, the virtual image is at near depth. On the contrary, when display panel is far from the curved mirror, the virtual image is at far depth. Part 2 and 3 show actuations of CASA with and without BPS in AR glasses.

Supplementary Movie 9

CASA in transparent haptic glove for visualization
The scalable CASAs are embedded in the wearable haptic glove. This video describes realizing tactile sensation by interaction between human skin and wearable haptic glove. Part 1 shows actuation mechanism of CASA in haptic glove and actuation of CASA in a fingertip. Part 2 shows simultaneous actuation of all CASAs in fingertip and palm of human hand.

Supplementary Movie 10

Sensing function of CASA in wearable haptic glove
This video shows the CASA can detect an external force. The CASAs embedded in haptic glove can be served as a force sensor. The resistance of SMA wire in CASA changes by contact force of the fingers.
